# Experiences, barriers and expectations regarding current patient monitoring systems among ICU nurses in a University Hospital in Lebanon: a qualitative study

**DOI:** 10.3389/fdgth.2024.1259409

**Published:** 2024-02-19

**Authors:** Ahmad Rayan, Suhair H. Al-Ghabeesh, Mirna Fawaz, Amal Behar, Amina Toumi

**Affiliations:** ^1^ Faculty of Nursing, Zarqa University, Zarqa, Jordan; ^2^ University of Business and Technology (UBT), Jeddah, Saudi Arabia; ^3^ Faculty of Nursing, Al-Zaytoonah University of Jordan, Amman, Jordan; ^4^ Department Faculty of Health Sciences, Beirut Arab University, Beirut, Lebanon; ^5^ Health Information Management Department, Liwa College of Technology, Abu Dhabi, United Arab Emirates

**Keywords:** monitoring systems, ICU nurses, experiences, barriers, expectations

## Abstract

**Purpose:**

The aim of the study is to assess the experiences, barriers, and expectations regarding current patient monitoring systems among intensive care unit nurses at one university hospital.

**Methods:**

A qualitative exploratory study approach was adopted to test the research questions.

**Results:**

Intensive care unit personnel placed a high value on practical criteria such as user friendliness and visualization while assessing the present monitoring system. Poor alarm handling was recognized as possible patient safety hazards. The necessity of high accessibility was highlighted once again for a prospective system; wireless, noninvasive, and interoperability of monitoring devices were requested; and smart phones for distant patient monitoring and alert management improvement were required.

**Conclusion:**

Core comments from ICU personnel are included in this qualitative research on patient monitoring. All national healthcare involved parties must focus more on user-derived insights to ensure a speedy and effective introduction of digital health technologies in the ICU. The findings from the alarm control or mobile device studies might be utilized to train ICU personnel to use new technology, minimize alarm fatigue, increase medical device accessibility, and develop interoperability standards in critical care practice.

## 1 Introduction

Critically ill patients must be closely monitored and treated precisely in Intensive Care Units (ICUs) as they require continuous medical attention (1). Patient monitoring systems, which provide real-time data on vital signs, physiological parameters, and alerts to enable prompt and accurate decision-making, are invaluable tools for ICU nurses in these high-stress conditions. Advanced patient monitoring systems are becoming increasingly prevalent in ICUs worldwide as technology develops continuously (2).

ICU nurse processes may be streamlined, clinical outcomes can be improved, and patient safety can be increased with the use of dependable and effective patient monitoring systems. They have the benefit of ongoing data collection, early condition detection, and customizable alarm settings according to patient requirements. Additionally, these systems can enhance coordinated treatment and interdisciplinary collaboration by facilitating the integration and exchange of patient data among healthcare practitioners (3).

However, there are challenges to overcome before patient monitoring systems may be proficiently utilized in ICU (4). ICU nurses are pioneers in the application of these technologies, and their insights and experiences are indispensable in identifying the benefits, drawbacks, and potential areas for development (5, 6). Furthermore, the efficacy and adoption of these technologies in the critical care context are significantly shaped by the experiences, barriers, and expectations of intensive care unit nurses with respect to currently available patient monitoring systems. ICU nurses have significant perspectives into the real-world applications of patient monitoring systems because they are frontline healthcare providers. Their experiences frequently demonstrate the advantages, which include enhanced patient safety, early identification of key events, and real-time data access (7). There are significant limitations to be addressed, nevertheless, such as alert fatigue brought on by a high number of false alarms, difficult user interfaces, difficulties with interoperability, and a lack of full integration with electronic health record systems (8). Additionally, according to Kannampallil and Adler-Milstein (9), ICU nurses anticipate better system use, streamlined workflows, individualized alarm settings, seamless data sharing, and greater interprofessional communication.

ICU nurses frequently use their personal experiences to emphasize the advantages of patient monitoring systems. By giving nurses access to continuous and real-time vital sign data, these systems enable them to closely monitor patients' physiological characteristics and quickly detect any deviations from the normal range. This makes it possible to identify deteriorating situations early on, which allows for prompt actions and could ultimately prevent unfavorable developments. ICU nurses value the efficiency and convenience that patient monitoring systems provide because they have instant access to patient data, which eliminates the need for manual charting and allows for tailored patient care (10).

Notwithstanding the benefits, there are significant challenges that affect how often and how well patient monitoring devices are used in intensive care units. One of the most prevalent barriers is alarm fatigue, as ICU nurses frequently deal with a large frequency of alerts, which can cause desensitization and the possibility of missing vital alarms. According to Lewandowska et al. (11), the intricacy of user interfaces and system navigation can present difficulties as well, necessitating more training and time to become proficient in using the systems. Problems with interoperability between various electronic health record systems and monitoring devices can impede the smooth integration of data, resulting in inconsistent patient information and possible errors. Furthermore, confusion and alert fatigue may result from disparities in alarm settings and thresholds among various devices (12).

ICU nurses seek that advances in patient monitoring systems will improve their usefulness and solve current issues. They frequently want workflows that are simplified and facilitate effective data entry and retrieval. Alert customization options are essential because they allow nurses to adjust thresholds according to the specific conditions of each patient, which lowers false alert rates and increases alarm precision. Efficient integration with electronic health record systems is highly regarded since it permits interdisciplinary contact and provides extensive patient data access. Additionally, ICU nurses expect enhanced communication capabilities from the technologies, like real-time notifications and alerts that make it easier to collaborate with other medical professionals punctually (13).

It takes a multifaceted approach to address these experiences, barriers, and expectations. It is a necessity for engineers, system developers, and healthcare practitioners to work together to provide user-friendly interfaces, enhance interoperability, and optimize alarm management techniques. To guarantee that ICU nurses are competent in using patient monitoring systems and handling alerts, training programs ought to be put in place. Standardization initiatives, such reaching an agreement on thresholds and settings for alarms, can lessen alarm fatigue and improve alarm precision. Furthermore, taking into account the input provided by ICU nurses during the design and development phases of patient monitoring systems will ensure that the technology aligns with their requirements and standards, which will eventually improve patient care and safety in the ICU (14).

Patient monitoring technologies in Lebanon have seen progressions in recent years, giving healthcare providers useful tools for patient care. Commonly utilized technology includes vital signs monitors, electrocardiogram (ECG) machines, and pulse oximeters, which enable continuous monitoring of patients' physiological indicators. These technologies afford real-time data and alerts to warn nurses of any alterations in patients' states, allowing for timely treatments and improving patient safety.

However, Lebanon might benefit from the implementation of developing skills that have the potential to simplify nurses' jobs and enhance patient outcomes. One such technology is remote patient observing, which allows healthcare personnel to monitor patients' vital signs and health state from a distance. This can be especially useful in cases where patients are discharged from the hospital but still need close monitoring. Remote monitoring can help minimize readmission amounts and allow nurses to respond early in the event of difficulties. Another new technology is wearable devices that measure numerous health metrics such as blood pressure, heart rate, and activity levels. These devices can offer continuous monitoring and create valuable, useful data for nurses to analyze patients' development and make educated decisions about their treatment. Furthermore, integrating artificial intelligence (AI) and machine learning systems into patient monitoring systems can assist evaluate massive volumes of data, detect trends, and forecast probable bad occurrences. This can help nurses' spot deterioration earlier and make proactive measures and interventions.

Adding mobile applications and cloud-based platforms to patient monitoring systems can also improve nurses' productivity and communication. These technologies provide seamless data exchange, remote access to patient data, and efficient cooperation among healthcare personnel. Additionally, smart alarms and sophisticated filtering algorithms can assist decrease alarm fatigue and enhance alert accuracy, ensuring that nurses get relevant and actionable messages. By adopting these developing technologies, Lebanon may improve the skills of its patient monitoring systems and empower nurses in their everyday job. The combination of remote monitoring, wearable devices, AI, and mobile applications can expedite processes, enhance patient outcomes, and eventually contribute to offering high standards healthcare services in the country.

This study aims to support ongoing efforts to optimize patient monitoring methods in critical care settings by illuminating the viewpoints of ICU nurses. In order to build and deploy patient monitoring systems that meet the requirements and expectations of ICU nurses, healthcare administrators, engineers, and legislators will find great value in the findings, which will eventually enhance patient care and safety in the ICU setting.

### 1.1 Research question

How do ICU nurses perceive their experience in handling current patient monitoring systems and what expectations do they have in the future?

## 2 Study design and method

### 2.1 Design

An exploratory qualitative phenomenological study research design was used. We relied on semi-structured interviews as the main basis for data collection so that it provides the researcher with a loose framework for undertaking interviews and motivates nurses to provide a rich account of their experiences that are considered relevant (15).

### 2.2 Setting

This study was carried out in ICU, at one university hospital in Lebanon. This hospital is designed to offer patients the most innovative treatments and technologies available in the region, in a safe and compassionate environment. The hospital, with 200 patient's beds, served by around 125 specialized doctors, 322 nurses, and 353 administrative employees, has been committed to making high-quality healthcare universally accessible in Lebanon.

### 2.3 Population and sampling

Nurses who served in the ICU of the approached hospital, were featured in the study's sample. The research used a convenience sample, which comprised all critical care nurses who had been practicing in the critical care area, satisfied the qualifying requirements, and volunteered to be included in the study. The researcher approached 30 ICU nurses, where 26 of them agreed to take part in the study yielding a response rate of 86.66%.

#### 2.3.1 Inclusion criteria

Nurses working in the ICU for more than six months were selected for this project so that they would have enough experience to effectively handle issues concerning monitoring systems and devices.

### 2.4 Instrumentation

#### 2.4.1 Demographic characteristics

A demographic data questionnaire was used to collect data about the nurses' age, gender, and years of experience, educational level, and marital status.

#### 2.4.2 Semi-structured interviews

After completing the demographic data sheet, the nurses who are willing to participate were invited to take part in semi-structured interviews which were employed to collect qualitative data regarding the experiences of those nurses. Possible respondents were approached by phone and the purpose of the research was clarified. Respondents were advised that participation would comprise of a recorded interview that would take about 1 h of their time to be conducted at a place of their choosing. The voluntary aspect of participation was emphasized, as was the freedom at any moment to refuse to answer a query or to end the conversation. The participants were advised that this conversation is completely anonymous and that any verbatim published would not be disclosed by name, but rather that information would be coded for privacy. Respondents were also advised that if they feel the need to withdraw from the study at any stage, they can. The conversations were recorded and interpreted in audio, but in order to mitigate any pitfalls that could be caused by the live recordings, the participants were informed that no one but the interviewer can listen to the live recordings and that it would be kept secure. Each interview lasted 20–30 min and covered all elements of the subject. This time was set aside to study all areas of the problem and to avoid leaving any blind spots. The interviews were conducted by one researcher trained in qualitative inquiry. At the outset of each conversation, the researcher introduces themselves and the conversation's purpose to the participants. After the respondents gave their verbal approval, the records were created. Each session lasted 20 min in total. In Arabic, open, non-directive questions were posed. The interviewer made sure to practice reflexivity during the interview process and took fields noted to capture any contextual and non-verbal cues that could help later in data analysis. Data collection extended between February 2022 and June 2022.

#### 2.4.3 Interview questions

The interview guide was developed based on the literature review conducted. Considering that this study relied on semi-structured interviews, the researchers intended to use multiple open ended questions rather than develop a thorough detailed interview guide to give space to more in-depth conversations and data grounded in the perspectives of the participants rather than enforced by the detailed interview guide. The questions were:

- How do you describe your experience in handling patient monitoring systems?
- How do you describe the challenges that you face with patient monitoring systems?
- How do you describe your expectations from technological advancements regarding patient monitoring systems?

If the interviewer needed more details or clarifications, they used probing questions such as “Can you explain more about this?” or “Can we emphasize on that thought?”. Further probing questions or prompts were inspired by sequential interviews through contestant comparative analysis.

### 2.5 Data analysis

The data was analyzed through a concurrent process of constant comparative analysis. The analysis was done by three different researchers, including the researcher who conducted the interviews. The use of investigator triangulation in data analysis helped ground the themes generated in the perspective of the participants provided in the transcripts. The different researchers conducting analysis convened after analyzing the data on their own and discussed the generated codes and themes until consensus was reached. This kept the researchers' bias in check and kept the findings grounded in the data. Constant comparative analysis was employed, where after each interview concluded, it was directly transcribed, and axial coding of the transcripts commenced the analysis of the collected information. This analysis informed the interviews in the further rounds. Each transcribed answer has been allocated to a category that matches the thought it conveys. These classifications were then broken into more general themes, which were then further subdivided into more particular subcategories based on the topic's specificity. The purpose of this concurrent, constant comparative analysis was to find commonalities that contrast, supplement, and support each other in the multiple interviews. NVivo was used to help in data analysis.

### 2.6 Trustworthiness of the study

Several procedures were undertaken to eliminate any restrictions that may have influenced the qualitative outcomes of this study. The results were then subjected to concurrent analysis to verify that the principles derived from the theme assessment remained relevant and correct within the context of the findings, conveying a clear sense of nurses' experiences. The researcher approached all of the participants on the same platform at a time that was convenient for them, and they were all addressed in the same courteous and friendly manner. In reality, the questions were asked in the same way, and the interviewer covered all relevant issues and did not leave any material out. The conclusions of the study were supported by clear verbatim quotations, giving the participants in this study a legitimate voice.

### 2.7 Ethical considerations

Ethical approval was sought from the Makassed General Hospital research ethics committee. The submitted document outlined key aspects of the research project covering its purpose, design, procedure, recruitment, participants' information, ethical underpinning, procedures for ensuring researcher safety, and risk evaluation involved in the hospital setting. The project only proceeded after approval was finally granted. Also, an informed consent from participating nurses was acquired in this study. The participants were no compensated considering that participation was upon convenience and the time engagement was no more than 30 min. Lastly, the anonymity and confidentiality of all participants in the study was ensured at all times during the research process.

## 3 Results

### 3.1 Sociodemographic data

The sample of this study comprised of 26 ICU nurses working at the approached university hospital, where 14 (53.84%) were female while 12 (46.15%) were males. The participants' mean age was 24.67 ± 2.18, where the median ICU experience among them was 6 years ranging from 1 year to 15 years. The nurses who took part in this study had various academic levels where 15 (57.69%) had a bachelor's degree while 11 (42.31%) held a master degree in critical care nursing.

### 3.2 Phenomenology

**Figure 1:**
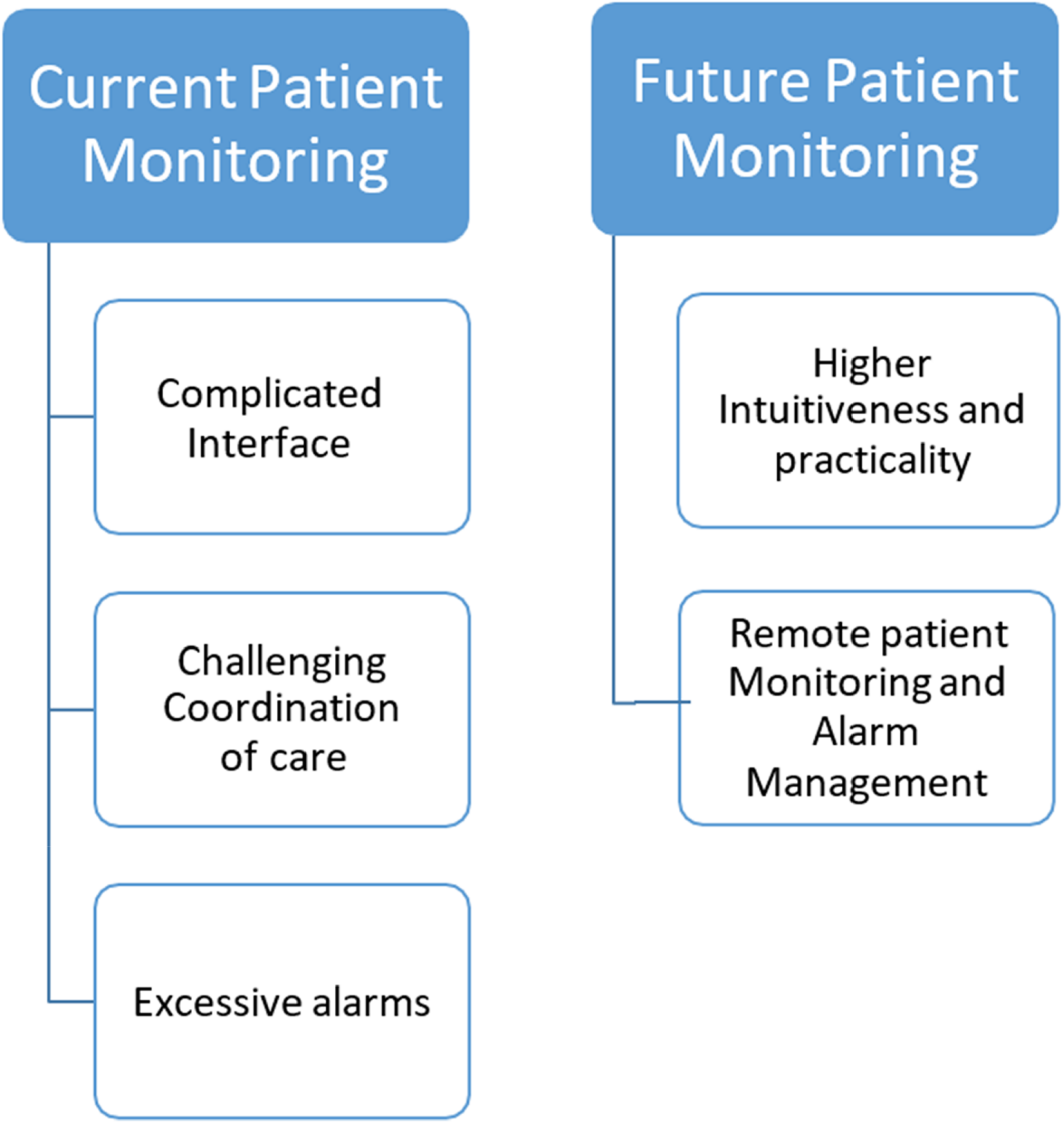
Thematic tree.

The thematic analysis gave rise to two major themes, namely “Current Patient Monitoring” and “Future Patient Monitoring” (Figure 1).

#### 3.2.1 Current patient monitoring

The first theme that emerged upon thematic analysis related to the experiences that were faced by the ICU nurses who participated in this study. These experiences were illustrated in three subthemes, namely, “Complicated Interface”, “Challenging coordination with care practices”, and “Excessive alarms”.

##### 3.2.1.1 Complicated interface

Upon thematic analysis, the researcher identified that the participating nurses recurrently referred to the complexity of the user interface of current patient monitoring systems, where they had to deal with a lot of tabs and settings and parameters to reach the desired monitoring outcomes especially when needed advanced monitoring. The present patient monitoring system's functionality was assessed as satisfactory by the ICU personnel who were questioned, with a focus on user friendliness and consistency. Standard functions such as vital parameter display and alert threshold setup were simple to use, but complex configurations were difficult to implement without education. For instance, one of the nurses proclaimed,

“…at first it was very hard for me to even know which button measures the blood pressure…or retakes the CVP or measures the SPO2…then I got the hang of it…however it is still very complicated to get to the advanced settings like changing an alarm threshold or setting up to PAWP wave…” (N2).

A comparable account was shared,

“…the way the monitor looks and the way it present all these data in a crowded interface really doesn’t not help me in reading the information and takes me a good minute to know at what I am looking…this does really lags me in an emergency… I mean I should be able to clearly identify what I am looking at in a very short moment…not look for the heart rate for a minute…” (N26).

##### 3.2.1.2 Challenging coordination with care practices

Another subtheme that was prevalent among the challenges experienced with the current patient monitoring systems related to the difficulty in visualizing the patient monitor while carrying out other care practices. The ICU nurses reported that the way the monitors are designed and the way the ICU is designed hinders them from carrying out care tasks while keeping an eye on essential monitor indicators. For instance, one of the nurses said,

“… I would be suctioning a patient and I need to monitor the blood pressure, the heart rate, the SPO2… but ergonomically I can’t easily do that I would have to twist and turn and uncomfortably carry out the suctioning so that I can see the monitor…” (N10).

A further account was shared,

“…being detached from the monitor for a long time while you do other tasks really puts you on your nerves especially with a high acuity patient were you would have to keep an eye out or when you are carrying out a hard task…I would be elbow deep in care and I would have to check on the monitor…. this is not comfortable …” (N5).

##### 3.2.1.3 Excessive alarms

Alarm maintenance was outlined the most when it came to patient monitoring aspects utilized by ICU personnel. Alarm levels would be adjusted on a routine basis by nurses based on existing patient status. Alarm exhaustion scenarios (i.e., numerous alarms ringing off at the same time) were identified as a key flaw in the present system, resulting in stress among patients and personnel, as well as a possible reduction in patient security. The following rationales were given: (1) operational: difficulty distinguishing among false and essential alarms, and vulnerability to inaccuracy of the Electrocardiogram, peripheral capillary oxygen saturation (SpO2), and end-tidal carbon dioxide (etCO2) detectors; (2) patient-related: intrusion of artefacts related to disorientation (such as excessive motion of patients), septicemia (for example centralized circulation), or high excessive sweating; and (3) ICU personnel: insufficient alarm hygiene owing to shortage of nurses.

For example one of the nurses said,

“In the ICU, alarm control is a major issue; some individuals set warning thresholds too high, which results in many false alerts. I believe it is critical to improve alarm handling among the staff, particularly in the evening, as well as the noise for the patients. When the client is meant to be sleeping and the monitor continues to bleep…” (N25).

Another nurse also proclaimed,

“Alarm hygiene is neglected much too often. This is not owing to professionals’ laziness, but rather to a staffing shortage; there are too little nursing staff and physicians. As a result, it simply pings a lot… Is it important or not that the monitoring system can't tell the difference? You won't glance at it the seventh time since you've got an alert 5 times since the patient is restless and hence the heart rate is presumably raised, but perhaps there’s anything else. Yes, this is an issue, because one serious circumstance or another is only discovered very late…” (N4).

#### 3.2.2 Future patient monitoring

The second major theme that was prevalent upon thematic analysis related to the expectations of the nurses from future advancements in patient monitoring systems. The theme comprised of three subthemes, “Higher intuitiveness and practicality”, and “Remote patient monitoring and alarm management”.

##### 3.2.2.1 Higher intuitiveness and practicality

Respondents stressed the relevance of a prospective patient monitoring system's user friendliness and usefulness, particularly in a crisis, as well as the potential to add more intricate and unique parameters. For instance, one of the nurses said,

“… if you're going to employ anything like this, it'd be better if it had more features and could be customized… Since, I reason, there are numerous distinct specialized teams moving around for example a senior specialist in the ICU may value characteristics that a nurse or another professional personnel does not..” (N1).

Another nurse also said about intuitiveness,

“In my opinion, everything should be self-explanatory seeing as we have far too many complex technologies to deal and take care of, therefore it would be ideal if everything was really user-friendly” (N22).

Nurses saw future models as more exact in measuring while also being less intrusive, portable, and with improved compatibility across medical equipment, such as accessibility to PDMS via patient monitors.

“With little work, acquire more data… So, certainly, it would be minimally intrusive and a bit more accurate …In any event, a cordless monitor communication would be fantastic. As this would, obviously, provide a significant functional benefit to the patient” (N14).

##### 3.2.2.2 Remote patient monitoring and alarm management

Respondents who participated in this study thought that employing mobile connection technologies, such as tablets or smartphones, as remote patient monitoring devices might enhance patient security, shorten ICU stays, and boost employee contentment. For instance, one of the nurses said,

“Using remote patient monitoring through handhelds and smart devices is without a doubt, a move in the correct route. Then again, it is beneficial to the patients. In the finest scenario, it gets the job simpler” (N6).

Another nurse also proclaimed,

“… we are used to using our phones rather than tablets as they are bulky to handle or to put in the uniform… it would be so natural for us to move to that direction where we can know what the patient is experience through a vibration in our smart watch for example…” (N12).

Improved alarm control, such as the ability to dismiss incorrect alerts from a mobile device and therefore reduced noise nuisance, was mentioned and supported as a way to reduce tension among both ICU personnel and clients via remote monitoring. For instance one of the nurses said,

“uhh… I suppose it would make my life simpler if I could dismiss some alerts while seated at my PC rather than needing to go to the centralized computer. Above all, it might keep the patient safe. You don't disregard erroneous alerts, or other warnings that you perceive as errors might be dangerous … and you don't think the client is less worried if he doesn't hear these sirens all the time in his own bed…I believe I'm also keeping psychosis at bay…” (N20).

Respondents also suggested an alert filtration mechanism run by the staff nurses and crucial alarm transfer to their smartphones to prevent nurse distractions caused by false alerts.

“…If you're always disturbed by other matters, I believe you'll do less in the time you possess. As a result, in response to your query, while it is critical that you be warned, excessive alerts must be filtered out in the end…” (N17).

## 4 Discussion

This qualitative research sheds light on the complexities of ICU patient surveillance. The present patient monitoring technology was simple to operate for vital sign surveillance by ICU personnel, but other aspects were challenging to set up owing to a shortage of education and a paucity of employees. Alarm exhaustion was cited as important risks to patient safety by ICU personnel. In the years ahead, a more compatible, accessible patient monitoring technology was required, with the ability to add sophisticated and customized features based on the demands of the patients or consumers.

Assessments and alerts for vital parameters should be more precise while being non-invasive and unobtrusive (e.g., wireless). Surprisingly, interviewers identified large-screen smart phones as a viable remote patient monitoring technology, which might minimize noise contamination, improve patient safety, and raise work satisfaction (16).

Despite the fact that alarm control is a key aspect of the patient monitoring systems utilized at the research site, there is presently no regular staff training or a structure for alarm administration in place. This is a disturbing discovery found in previous research and consistent with our study where in a scenario when “cry wolf” situations with many alarms going off at the same time have become the norm in the ICU has been prevalent. Up to 99 percent of all audio alerts have been characterized as false alarms that do not modify patient therapy (17). The patient's health, the users' competency, and the technological elements of the patient monitoring system all play a role in these near misses. False alarms de-sensitize healthcare personnel to crucial alerts and are a serious patient safety concern, with thousands of patients dying as a result of false alarms each year (18). Per the findings of our study, patient safety may be jeopardized as a result of persistent noise pollution, which causes disruptions, tension, and attention problems among ICU staff. Although numerous techniques for reducing false alarms in the ICU have been established, their adoption into a clinical practice is still inadequate. Alarm management tactics reduce alerts by 24 percent to 88.5 percent per ICU, demonstrating the efficiency of such measures, which include staff training for each ward that incorporates patient monitoring devices (19).

The majority of acute-care medical equipment are not intended to work together nowadays. Surprisingly, our findings show that future patient monitoring expectations are progressively expanding to include more than just critical indicator monitoring. As previously proposed by Nemati et al. (20), ICU personnel want a patient monitoring device that can interconnect with various medical equipment for extensive evaluations of vital indicators and pattern analysis in the context of medicine, ventilation, fluid balance, and more. In the ICU, this might improve productivity and decrease unnecessary paperwork. In terms of convenience, ICU personnel in our study showed a need for clinical solutions that are intuitive and responsive. Despite the fact that computer applications in health care have been in use for more than a decade, usability—the capacity to utilize technologies in a fast, productive, and secure manner not yet been fully tuned for clinical use. Digital apps should not cause stress in the ICU. Rather, their usage should direct the consumer to perform work that is efficient, effective, and safe. Various basic and low-cost approaches for usability study are accessible, and everyone engaged in medical device development should use them (21).

Hospital settings have benefited from the usage of tablet devices with access to patient health records or potentiometric surveillance. Larger tablets, on the other hand, were too big to carry about owing to the limited compartments of ICU staff's uniforms as indicated in our study; rather, they chose small portable tablets or bigger mobile phones for patient monitoring in the Intensive care unit. This discovery might have an impact on future device design for the ICU and operating theatre, where uniforms are worn. Foldable phones, which were recently debuted, might be a way to combine the benefits of pocket-sized and large-screen smartphones (22). More multidisciplinary research is needed to collect early input from doctors, programmers, and designers, as industry players are already building apps for mobile smartphones in the Intensive care unit. The mobile smartphone or tablet might readily be employed for these activities in the trend toward wider deployment of telemedicine and remote patient monitoring technologies in different health care sectors, such as the ICU. The use of remote patient monitoring, according to ICU personnel, might minimize the length of stay in the ICU, which is in accordance with other recent research on telemedicine (23).

### 4.1 Limitations and implications

One of the main limitations of the study is that it focuses on nurses in one University Hospital in Lebanon. As a result, the findings might be context-specific and may not be applicable to other healthcare settings or regions. The study, however, can provide valuable insights for hospital administrators, policymakers, and healthcare practitioners to understand the challenges and expectations related to patient monitoring systems among ICU nurses. The findings can be used to enhance the quality of care and the working conditions of ICU nurses. Moving forward, we aim to collaborate with researchers from different countries to conduct cross-cultural studies, considering variations in healthcare systems and practices. In addition, we aim to implement and evaluate interventions based on the identified barriers and expectations to assess their impact on nurse satisfaction and patient outcomes.

## 5 Conclusion

Health care stakeholders may need to concentrate more on user-derived discoveries than top-down predictions in order to adopt more durable digital health technologies in the Intensive care unit. We may acquire health care professionals' trust by appreciating their ideas and implementing revolutionary methods by respecting their perspectives. The findings on alarm administration and portable devices in the ICU, in particular, could be used by healthcare institutions to brace ICU employees for digitalization, research facilities to decrease alarm fatigue, industry leaders to accept medical device functionality, and governmental organizations and decision makers to advance interoperability standards in critical care medicine. Other academics should be inspired by your results to perform qualitative patient- and user-centered studies in health care, particularly before inventing or applying hasty technical solutions.

## Data Availability

The original contributions presented in the study are included in the article/Supplementary Material, further inquiries can be directed to the corresponding author.
